# Complicated appendicitis increases the hospital length of stay

**DOI:** 10.1016/j.sopen.2022.05.006

**Published:** 2022-05-20

**Authors:** Abdulrahman Muaod Alotaibi, Mohammed Alfawaz, Lina Felemban, Leena Moshref, Rana Moshref

**Affiliations:** a Department of Surgery, Faculty of Medicine, University of Jeddah, Jeddah, Saudi Arabia; b Department of Medicine, Faculty of Medicine, University of Jeddah, Jeddah, Saudi Arabia; c Department of Surgery, King Abdul-Aziz Medical City, National Guard Hospital, Jeddah, Saudi Arabia; d Department of Surgery, Dr Soliman Fakeeh Hospital, Jeddah, Saudi Arabia

## Abstract

**Background:**

There are insufficient data from Saudi Arabia regarding appendectomy outcomes and hospital length of stay. Further, there is a need to compare the length of stay of Saudi patients and the literature. The purpose is to evaluate the surgical outcomes and hospital length of stay for complicated appendicitis and simple appendicitis.

**Method:**

This is a single-center retrospective review of patients who had undergone an appendectomy between 2016 and 2018. The patients were divided into 2 groups: complicated appendicitis versus simple appendicitis.

**Results:**

Of 449 patients who underwent appendectomy, 60 (13.4%) had complicated appendicitis. The complicated appendicitis was significantly associated with increased age, pain duration, neutrophilia, high C-reactive protein, fecalith presence, and free fluid. The incidence rate of surgical site infection was 5.8% (identified in 26 patients). Compared to simple appendicitis, complicated appendicitis was associated more with wound infection (1.8% vs 10%, respectively, P = .001), postoperative collection (1.2% vs 11.6%, respectively, P = .001), and readmission within 30 days (2.3% vs 13.4%, respectively, P = .001). By multivariate analysis, factors associated more with increased hospitalization were pain duration (hazard ratio = 2.37, 95% confidence interval = 1.09–5.16, P = .029), operative time (hazard ratio = 2.09, 95% confidence interval = 1.04–4.21, P = .038), and complicated appendicitis (hazard ratio = 6.61, 95% confidence interval = 2.67–14.21, P = .001).

**Conclusion:**

Complicated appendicitis correlates with significant morbidity, readmission rate, and 6 times more hospital LOS than simple appendicitis. This review might help in appreciating the burden of complicated appendicitis on hospital length of stay, which needs allocating patients and planning the discharge day for hospitals with limited beds.

## Highlights

• Practically one fifth of acute appendicitis will be complicated.
• Complicated appendicitis increases conversion to open, surgical site infection, hospital length of stay, and 30-day readmission rate.
• Prolonged operation time and complicated appendicitis are independent risk factors to increase hospitalization.

## INTRODUCTION

Acute appendicitis (AA) has a lifetime risk of 8.6% and 6.7% in males versus females, respectively, tending toward young adults with lower abdominal pain [1,2]. Complicated appendicitis (CA) has a delayed presentation with predictable risk factors comprising age > 50 years, female sex, symptoms of 2 days, elevated Alvarado score, C-reactive protein (CRP) > 100 mg/L [[3], [4], [5], [6]], and high infection rate postoperatively in diabetic patients [7]. More than 300,000 appendectomies are performed annually; 20% are complicated with diffuse peritonitis, perforation, abscess, and phlegmon [8]. With a delay in seeking medical attention, the need for early diagnosis and management in CA remains challenging and controversial [9,10]. A meta-analysis revealed that an approach to CA by laparoscopic access is widely accepted as it decreases the infection rate and hospital length of stay (LOS) [11,12]. Additionally, there was no significant difference in intra-abdominal abscess rates compared to open groups [12].

There is a data insufficiency from Saudi Arabia regarding appendectomy complications, outcome, and hospital LOS. Further, there is a need to highlight the LOS of Saudi patients compared to the international figures [13]. This review explores the influence of CA in delaying patient discharges. The tested hypothesis is that CA is associated with more than 72 hours of hospitalization than simple appendicitis.

## METHODS

The appendectomies' surgery evaluation and data collection were retrospectively performed at a single, private tertiary center, Dr Soliman Fakeeh Hospital (Jeddah, Saudi Arabia), between January 2016 and December 2018. We excluded pediatric age groups, patients who had an appendectomy combined with other surgeries, and those who were managed conservatively. The *pediatric age group* is defined in our center as 14 years old. The patients were classified into complicated and noncomplicated groups based on their clinical, radiological, and intraoperative findings.

*CA* is defined as a perforated or gangrenous appendix. The diagnosis of CA was confirmed clinically and radiologically by ultrasonography, computed tomography (CT), and diagnostic laparoscopy. The primary outcomes were surgical approaches and postoperative complication rates. The secondary outcomes were the postoperative LOS and 30-day readmission rates. *Postoperative LOS* is defined as the duration between leaving the recovery room and discharge time recorded in hours. *Operative time* was defined as the time from skin incision to the application of the wound dressing [14].

The Institutional Review Board approved the study protocol of Dr Soliman Fakeeh Hospital, with approval no. 200/IRB/2021.

### Surgery Techniques

Laparoscopic appendectomy (LA) is the procedure of choice in our institution to manage AA. However, deciding to do an open appendectomy or convert from laparoscopy depends on the surgeon's preference and clinical condition. Four qualified consultant-level surgeons have performed the surgeries since the residency training program was initiated in 2019.

### Statistical Analyses

The demographic and clinicopathological variables of the 2 groups were compared using Fisher exact test with two-sided verification and Pearson *χ*^2^ test or an unpaired Student *t* test, depending on the nature of the data. In addition, multivariate logistic regression of the factors that increase the hospitalization time was conducted. Data were analyzed using SPSS software (version 25, SPSS Inc, Chicago, IL).

## RESULTS

### Characteristics of CA and SA groups

Of the 449 patients who underwent appendectomy, 60 had CA (13.4%). The mean age of the complicated group was 34.0 ± 13.2 years compared to 30.0 ± 12.1 years for the SA group (*P* = .019). Sex, diabetes, and other comorbidities, according to the American Society of Anesthesiologists (ASA) physical status categorization system, were not significant factors between both groups. The length of admission was significantly higher in the CA, with an average of 4.7 ± 3.4 days (range 1–28 days). The CA patients presented late compared to SA (60% vs 11.1%, *P* = .001). In addition, the laboratory finding was significantly higher in the CA with white blood cell (WBC) count average of 15.9 ± 6.6 × 10^3^/mm^3^ (range 3–50 × 10^3^/mm^3^), neutrophilia average of 78.84% ± 10.62%, and C-reactive protein (CRP) of 133.76 ± 103.4 mg/L (range 1–362 mg/L, *P =* .001) (Table 1).

**Table 1:** Demographics and clinicopathological features of the study patients according to complicated status

	*Values as mean ± SD or no. of patients (%)*		
*Variables*	*SA group*	*CA group*	P *value*⁎
	(*n* = 389)	(*n* = 60)	
Age at surgery, y	30 ± 12.1	34 ± 13.2	.019
Sex			.092
Male	218 (56)	41 (68.3)	
Female	171 (44)	19 (31.7)	
ASA score			.103
I + II	379 (97.4)	56 (93.3)	
III + IV	10 (2.6)	4 (6.7)	
Length of admission	2 ± 1.7	4.7 ± 3.4	.001
Diabetes			.59
Yes	21 (5.4)	3 (5)	
No	368 (94.6)	57 (95)	
Comorbidities			.29
Yes	69 (17.7)	14 (23.3)	
No	320 (82.3)	46 (76.7)	
Pain duration			.001
Less than 48 h	346 (88.9)	24 (40)	
More than 48 h	43 (11.1)	36 (60)	
WBC (10^3^/mm^3^)	12.11 ± 4.2	15.90 ± 6.6	.001
Neutrophil %	71.59 ± 13.7	78.84 ± 10.62	.001
CRP (mg/L)	35.90 ± 52.6	133.76 ± 103.4	.001
Type of radiology study			.38
Ultrasonography	149 (38.7)	19 (32.2)	
CT scan	240 (61.3)	41 (67.8)	
Appendix diameter (mm)	10.73 ± 3.95	13.41 ± 4.16	.001
Fecalith			.001
Yes	57 (14.8)	22 (38.6)	
No	327 (85.2)	35 (61.4)	
Fat stranding			.146
Yes	227 (59.1)	41 (68.3)	
No	157 (40.9)	19 (31.7)	
Free fluid			.001
Yes	88 (22.9)	27 (47.4)	
No	296 (77.1)	30 (52.6)	
Operative approach			.001
Open	43 (11.1)	13 (21.7)	
Laparoscopy	338 (86.9)	34 (56.6)	
Lap converted to open	8 (2)	13 (21.7)	
Operative time (min)	46 ± 17.5	77 ± 26.6	.001
Appendix stump closing technique			
Endoloop	302 (77.6)	23 (38.3)	
Stapler	36 (9.3)	11 (18.3)	.001
Suture	51 (13.1)	26 (43.4)	
Intraoperative finding			.001
Normal appendix	16 (4.1)	0	
Inflamed	373 (95.9)	0	
Gangrenous	0	7 (11.7)	
Perforated	0	39 (65)	
Mass	0	8 (13.4)	
Abscess	0	5 (8.3)	
Mucocele	0	1 (1.6)	
Peritoneal fluid culture			.001
Positive	30 (7.7)	28 (46.6)	
Negative/not done	359 (92.3)	32 (53.4)	
Isolated organism (positive culture)			
*E coli*	15 (50)	15 (53.5)	
ESBL	6 (20)	11 (39.2)	.001
Others	9 (30)	2 (7.3)	
Postoperative complication			.001
No	373 (95.9)	43 (71.6)	
Wound infection	7 (1.8)	6 (10)	
Collection	5 (1.2)	7 (11.6)	
Nonsurgical	4 (1.1)	4 (6.8)	
Pathology			
No suppuration	67 (17.3)	0	
Acute suppuration	304 (78.1)	3 (5)	
Gangrenous	0	15 (25)	
Perforated	0	37 (61.7)	.001
Endometriosis	1 (0.25)	0	
Carcinoid	3 (0.77)	0	
Chronic appendicitis	12 (3)	2 (3.3)	
Granulomatous	1 (0.25)	2 (3.3)	
Mucocele	1 (0.25)	1 (1.7)	
30-d readmission			.001
Yes	9 (2.3)	8 (13.4)	
No	380 (97.6)	52 (86.6)	
30-d mortality	0	0	

⁎ Pearson *χ*^2^ test or Fisher exact test.

Most of the patients in both groups underwent ultrasound or CT scans during evaluation as the images of choice. The diameter of the appendix tends to be more prominent in the CA (13.41 ± 4.16 mm, *P* = .001). Fecalith was associated more with CA (38.6% vs 14.8%, *P =* .001). The free fluid on images was almost 2 times higher in the CA (47.4% vs 22.9%, *P* = .001).

### Operative Result

CA group had higher rate of open appendectomy (21.7% vs 11.1%, *P =* .001), conversion rate (21.7% vs 2%, *P =* .001), and using the stapler device to close the appendix stump (18.3% vs 9.3%, *P* = .001). The reported intraoperative finding of CA was perforated 65%, mass 13.4%, gangrenous 11.7%, abscess 8.3%, and mucocele 1.6%. Positive peritoneal fluid culture presents more in the CA (46.6% vs 7.7%, *P =* .001), with *Escherichia coli* more frequently isolated in 53.5% followed by extended spectrum beta-lactamase (ESBL) in 39.2% (Table 1).

### Histopathology Result

**Fig 1:**
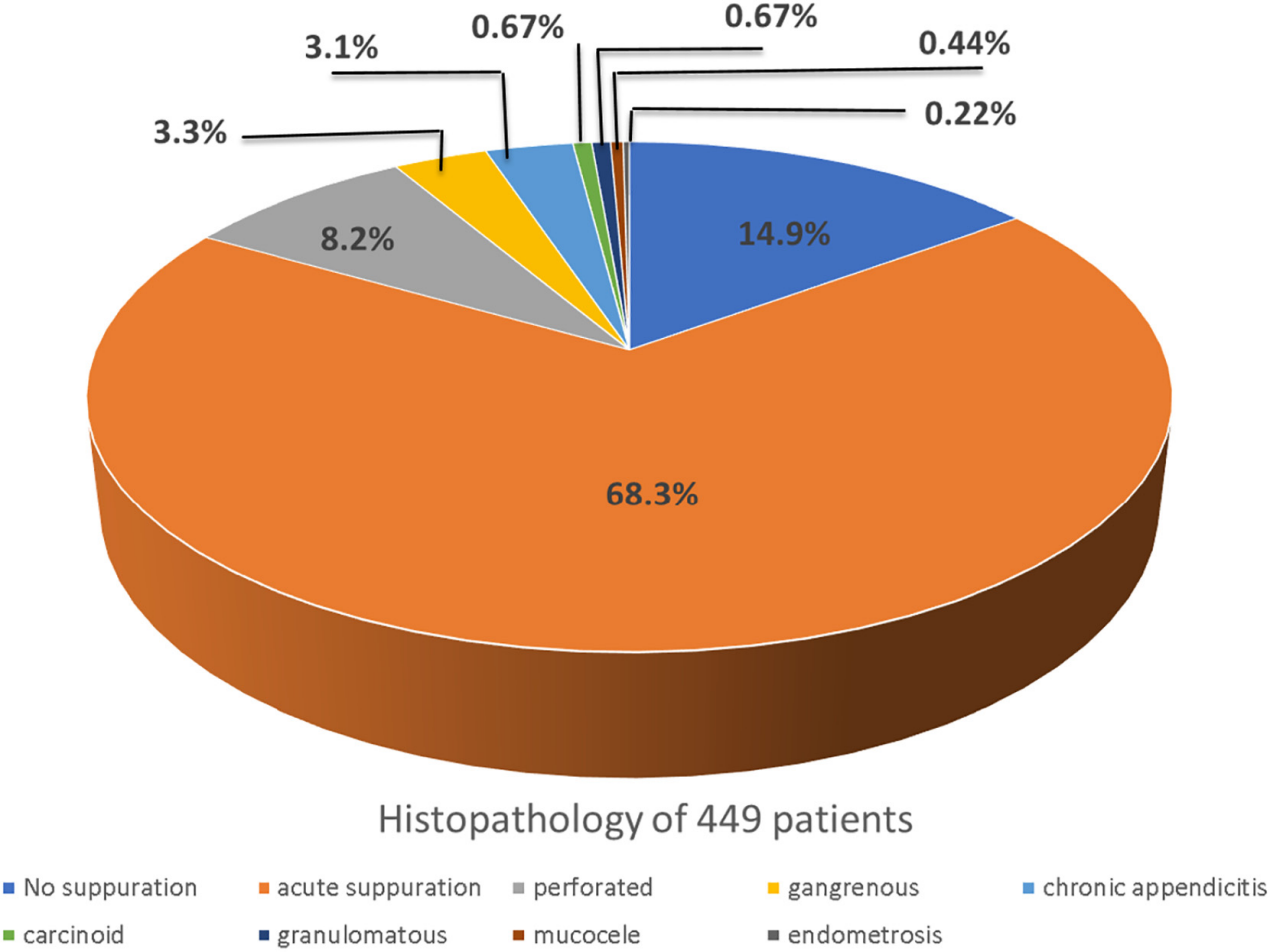
Pie chart demonstrates histopathology results of 449 appendectomies.

Among 449 patients, 14.9% (*n* = 67) were found to have nonsuppurative appendicitis, 68.3% (*n* = 307) had acute suppurative appendicitis, 8.2% (*n* = 37) perforated, 3.3% (*n* = 15) gangrenous, 3.1% (*n* = 14) chronic appendicitis, 0.67% (*n* = 3) carcinoid tumor, 0.67% (*n* = 3) granulomatous appendicitis, 0.44% (*n* = 2) mucocele, and 0.22% (*n* = 1) endometriosis-induced appendicitis (Fig 1).

### Postoperative Complications and Readmission

The incidence rate of surgical site infection (SSI) was 5.8% (identified in 26 patients). The CA compared to SA was associated more with wound infection (10% vs 1.8%, respectively, *P =* .001), postoperative collection (11.6% vs 1.2%, respectively, *P =* .001), and non–surgical-related complication (6.8% vs 1.1%, respectively, *P* = .001), mainly postoperative ileus in 5 patients (1.1%), and incisional hernia in 3 patients (0.67%).

The 30-day readmission rate was also significantly higher among CA (13.4% vs 2.3%, respectively, *P =* .001) (Table 1).

### Factors That Increase Hospitalization Time

Patients with CA were 6 times more likely to stay in the hospital for more than 72 hours. Factors associated with long stay include preoperative pain of more than 48 hours, appendix diameter of more than 13 mm, fecalith, free fluid, operative time of more than 60 minutes, high WBC, high CRP, and CA. Through multivariate analysis, the factors associated more with increased hospitalization were duration of pain (hazard ratio [HR] = 2.37, 95% confidence interval [CI] = 1.09–5.16, *P =* .029), operative time (HR = 2.09, 95% CI = 1.04–4.21, *P =* .038), and CA (HR = 6.61, 95% CI = 2.67–14.21, *P =* .001) (Table 2).

**Table 2:** Multivariate analysis of variables associated with more than 72 h hospitalization

*Variables*	*Univariate*	*Multivariate*		
	P *value*⁎	*HR*	*95% CI*	P *value*†
Pain duration	.001			.029
< 48 h		1		
≥ 48 h		2.37	(1.09–5.16)	
Diameter (mm)	.001			.29
Fecalith	.014			.90
Free fluid	.025			.77
Duration of surgery	.001			.038
< 60 min		1		
≥ 60 min		2.09	(1.04–4.21)	
Operative approach	.001			.29
WBC (10^3^/mm^3^)	.021			.64
CRP (mg/L)	.001			.81
Complicated appendicitis	.001			.001
No		1		
Yes		6.61	(2.67–14.21)	

⁎ Pearson *χ*^2^ test or Fisher exact test.

† Logistic regression test.

## DISCUSSION

Our review yielded that 13.4% of those who underwent appendectomy had CA. The incidence rate of CA can reach up to 25% [2]. In concurrence with our data, many authors confirm that ASA, diabetes, and comorbidities did not differ significantly between the 2 groups, with diabetic patients more prone to developing perforations and increasing hospital LOS [7,15].

Laboratory tests for CA patients show high WBCs, CRP, and neutrophilia. CRP, WBCs, and bilirubin were predictors of CA [6,[16], [17], [18]]. The same finding was seen in our patients; the average CRP of CA was 133 mg/L, and 78% had neutrophilia.

Ultrasound and CT scans have no statistical significance difference when diagnosing appendicitis [[19], [20], [21]]. A meta-analysis showed that preoperative CT reduced the rate of negative appendectomies to less than 10% but increased the time to surgery [18]. The radiological findings of enlarged appendix diameter, fecalith, surrounding fat stranding, and perforation were shown to be markers that predict CA [22]. Furthermore, periappendiceal fat stranding was the sole CT scan feature with 95% sensitivity yet low specificity of 40% [23]. In our imaging, CA is associated more with prominent diameter and fecalith. In addition, the amount of free fluid was nearly twice as high in the complicated group.

Laparoscopy is the prevailing method to remove the appendix [23]. CA has no standardized approach when associated with perforation (local/contained), abscess, or mass [24]. However, studies have shown that it is a safe technique to be performed in CA because the surgeon's preference and experience play a role in selecting this modality [12]. The reported intraoperative findings of CA in our article were perforation, mass, gangrenous, abscess, and mucocele with 65%, 13.4%, 11.7%, 8.3%, and 1.6%, respectively. The conversion rate was 2% in the SA group versus 21% in CA. One review reported 11% as the average conversion rate, which depends most of the time on surgeon experience and patient factors [25].

Conservative therapy with antibiotics is frequently implemented in uncomplicated presentation and has a success rate of approximately 2/3 and recurrence in 1/2 of the cases within 1 year. With antibiotic resistance on the rise, appendectomy remains the best treatment option for appendicitis [26]. We exclude the conservative management from the analysis because we are discussing the surgical outcome. In 1 local review of 327 patients, antibiotics showed a success rate of 88% at 1 year [27].

A meta-analysis of 67 trials promoted that wound infections are one half less in LA than in open (OR 0.43; 95% CI 0.34–0.54); on the contrary, intra-abdominal abscesses were 3 times higher in the LA group [27,28]. We noticed a collection rate of 2.2% after LA, but it was not statistically significant. LA had a shorter hospitalization and a faster recovery according to studies published in the last 30 years [18,[28], [29], [30]].

In one local review, SSI postappendectomy was found in 31 patients out of 433 (7%) [31]. Our data showed SSI in 26 patients (5.8%) and deep-spaced collection identified in 13 patients (2.9%) managed by CT-guided drainage. The high rates of postoperative wound infection and collection were associated more with *E coli* and ESBL isolation from the peritoneal fluid culture. Boueil et al found that nearly three quarters of people with ruptured appendicitis had positive peritoneal culture. *E coli* was the most prevalent organism [32]. Prior research has thoroughly investigated the 30-day readmission rates. CA affects the readmission with a lower rate in laparoscopic compared to open technique [33,34]. A recent meta-analysis substantiates that factors such as CA, diabetes mellitus, and open technique influence unexpected readmission within 30 days [35].

Another local review underlines the histopathology from 480 patients; it was appendicitis in 250 (52.0%), suppurative appendicitis in 135 (28.0%), acute gangrenous appendicitis in 60 (12.5%), perforated appendicitis 9 (2.0%), and chronic appendicitis 12 (2.5%). [36]. On the contrary, our report found an 8.2% perforation which can be deduced by the high rate of fecalith (38%) in the CA group.

The hospital LOS has considerably influenced the health insurance cost. Factors associated with increased hospitalization on multivariate analysis were the duration of pain, operative time, and CA. The complicated group has 6 times more prolonged hospitalization than 72 hours. It required an average of 3.56 postoperative days in 1 review of 186 patients with CA. [37] A 2012 retrospective analysis found that a longer operating time increases the hospital stay length when treating CA with a minimally invasive approach. Nevertheless, it has been linked to reducing postoperative morbidities and wound infection and allows a quick return to normal activities [35,38].

This framework, however, pertains to our center experience; its drawback is that an observational study with physician bias might exist in choosing the management approach. Despite that, it strengthens the association of CA with significant morbidity, postoperative complication, hospitalization, and readmission rate. This review might help in appreciating the burden of CA on hospital LOS, which needs allocating patients and planning the discharge day for hospitals with limited beds.

## Author Contribution

Abdulrahman Muaod Alotaibi made substantial contributions to the study's conception and design, data acquisition, analysis and interpretation, drafting of critical manuscript revisions, and approving the final version of the text as the corresponding author.

Lina Felemban helped with the literature review, wrote the manuscript, and approved the final version of the text.

Leena Moshref helped with the literature review, wrote the manuscript, and approved the final version of the text.

Rana Moshref helped with the literature review, wrote the manuscript, and approved the final version of the text.

## Conflict of Interest

The authors declare no competing interests.

## Funding Sources

The authors report no sources of funding for this article.

## Ethics Approval

The Institutional Review Board approved the study protocol of Dr Soliman Fakeeh Hospital, with approval no. 200/IRB/2021.
